# From Acute Carditis, Rheumatic Carditis, and Morphologic Cardiac Reactions to Allergic Angina, Allergic Myocardial Infarction, and Kounis Syndrome: A Multidisciplinary and Multisystem Disease

**DOI:** 10.3390/jcdd12090325

**Published:** 2025-08-25

**Authors:** Nicholas G. Kounis, Alexandros Stefanidis, Ming-Yow Hung, Uğur Özkan, Cesare de Gregorio, Alexandr Ceasovschih, Virginia Mplani, Christos Gogos, Stelios F. Assimakopoulos, Christodoulos Chatzigrigoriadis, Panagiotis Plotas, Periklis Dousdampanis, Sophia N. Kouni, Grigorios Tsigkas, Nicholas Patsouras, Gianfranco Calogiuri, Soheila Pourmasumi, Ioanna Koniari

**Affiliations:** 1 Department of Medicine, Division of Cardiology, University Hospital of Patras, 26500 Patras, Greece; gregtsig@upatras.gr (G.T.); npatsouras@gmail.com (N.P.); iokoniari@yahoo.gr (I.K.); 2 First Cardiology Department, General Hospital of Nikea, Agios Panteleimon Piraeus, 3 D Mantouvalou Street, 18454 Piraeus, Greece; plato203@yahoo.com; 3 Division of Cardiology, Department of Internal Medicine, Shuang Ho Hospital, Taipei Medical University, 18 No. 291, Zhongzheng Rd., Zhonghe District, New Taipei City 23561, Taiwan; myhung6@ms77.hinet.net; 4 Taipei Heart Institute, Taipei Medical University, Taipei City 110301, Taiwan; 5 Division of Cardiology, Department of Internal Medicine, School of Medicine, College of Medicine, Taipei Medical University, Taipei City 110301, Taiwan; 6 Department of Cardiology, School of Medicine, Trakya University, 22030 Edirne, Turkey; drugurozkan@hotmail.com; 7 Department of Clinical and Experimental Medicine, University of Messina Medical School, 98122 Messina, Italy; cesare.degregorio@unime.it; 8 Faculty of Medicine, “Grigore T. Popa” University of Medicine and Pharmacy, 700115 Iasi, Romania; alexandr.ceasovschih@yahoo.com; 9 Second Internal Medicine Department, “Sf. Spiridon” Clinical Emergency Hospital, 700111 Iasi, Romania; 10 Intensive Care Unit, Patras University Hospital, 26504 Patras, Greece; virginiamplani@yahoo.gr; 11 Department of Cardiology, Mamatseio, General Hospital of Kozani, 50100 Kozani, Greece; gogos-grivas@hotmail.com; 12 First University Cardiology Department, AHEPA General Hospital, Aristotle University of Thessaloniki, 54124 Thessaloniki, Greece; 13 Division of Infectious Diseases, Department of Internal Medicine, Medical School, University of Patras, University Hospital of Patras, 26504 Patras, Greece; sassim@upatras.gr; 14 School of Medicine, University of Patras, 26504 Patras, Greece; up1084142@ac.upatras.gr; 15 Department of Speech Therapy, University of Patras, 26504 Patras, Greece; pplotas@upatras.gr; 16 Department of Nephrology, Saint Andrews State General Hospital, 26221 Patras, Greece; dousdampanis@yahoo.gr; 17 Speech Therapy Practice, Queen Olgas Square, 26221 Patras, Greece; snkouni@yahoo.gr; 18 Department of Internal Medicine, Immunology and Infectious Diseases, Section of Allergology and Clinical Immunology, University of Bari Medical School, 70121 Bari, Italy; gf.calogiuri@libero.it; 19 Clinical Research Development Unit, Ali-Ibn Abi-Talib Hospital, Rafsanjan University of Medical Sciences, Rafsanjan 7717933777, Iran; spourmasumi@yahoo.com

**Keywords:** allergic angina, allergic myocardial infarction, COVID-19, degranulation, immunoglobulin E, Kounis syndrome, mast cell, tryptase

## Abstract

This narrative review explains the history of anaphylactic or hypersensitivity reactions, their connection to the cardiovascular system, and Kounis syndrome, which is linked to hypersensitivity. Additional subjects discussed include immunoglobulin E and serum tryptase, common pathways of allergic and nonallergic cardiovascular events, current perspectives on Kounis syndrome, allergic myocardial infarction, allergic angina, and the impact of COVID-19 and its vaccination on Kounis syndrome. Kounis syndrome is a distinct kind of acute vascular disease that affects the coronary, cerebral, mesenteric, peripheral, and venous systems. Kounis syndrome is currently used to describe coronary symptoms linked to disorders involving mast cell activation and inflammatory cell interactions, such as those involving T-lymphocytes and macrophages, which further induce allergic, hypersensitive, anaphylactic, or anaphylactic insults. Platelet activating factor, histamine, neutral proteases like tryptase and chymase, arachidonic acid products, and a range of cytokines and chemokines released during the activation process are among the inflammatory mediators that cause it. Proinflammatory cytokines are primarily produced by mast cells in COVID-19 infections. Mast cell-derived proteases and eosinophil-associated mediators are also more prevalent in the lung tissues and sera of COVID-19 patients. As a modern global threat to civilization, COVID-19 is linked to chemical patterns that can activate mast cells; therefore, allergic stimuli are usually the reason. Virus-associated molecular patterns can activate mast cells, but allergic triggers are typically the cause. By activating SARS-CoV-2 and other toll-like receptors, a variety of proinflammatory mediators, including IL-6 and IL-1β, are released, potentially contributing to the pathology of COVID-19.

## 1. Introduction

Pharaoh Menes passed away unexpectedly while visiting the British Isles some 4500 years ago, or in the year 2600 BC; a wasp or hornet sting was blamed for his demise. The hieroglyphs of two nearly identical ebony plates, albeit only partially preserved, discovered at one of the numerous suspected Menes burial sites serve as the basis for this event [1].

Approximately a century ago, the medical literature in English [2,3,4], German [5], and Austrian [6] began to feature cardiovascular symptoms and signs linked to allergic, hypersensitive, anaphylactic, or anaphylactoid reactions.

These reactions were identified as “morphologic cardiac reactions”, “acute carditis”, and “lesions with basic characteristics of rheumatic carditis”. They were primarily caused by serum sickness and tetanus antitoxin. Specifically, animal experimental models showed electrocardiographic alterations in dogs and rabbits but not in guinea pigs, as the latter typically perished during the experiments from asphyxia-induced death [7]. Today, certain inflammatory agents such as mast cells are triggered and connected to anaphylactic or anaphylactoid insults, as well as allergic or hypersensitive reactions [8]. These disorders involve other inflammatory cells that are related to and interact with each other, such as T-lymphocytes, macrophages, and mast cells. A number of mediators that are responsible for the allergic reaction are released by all of these inflammatory cells. Both high- and low-affinity immunoglobulin E (IgE) receptors are present on the surface of 20% of platelets, which aids in the allergic reaction. These inflammatory cells form a vicious cycle and activate one another like a ball of thread in response to different stimuli.

In particular, mast cells originate as mononuclear cell precursors from the bone marrow, circulate as mast cell precursors, and have KIT receptors for stem cell factor (SCF) on their surface [9]. One important cytokine that is necessary for mast cell development, growth, adhesion, proliferation, survival, and homing is SCF. Since IgE antibodies are unable to cross the blood–brain barrier, they spread to all human tissues, including the brain, which is immune to allergic reactions. Mast cells differentiate and mature there. This takes days, if not weeks, to happen. In contrast, basophils develop from granulocyte precursors in the bone marrow and enter the circulation as mature cells; they do not enter the tissues until the later stages of an allergic reaction.

In this review, we provide a description of the history of anaphylactic or hypersensitivity reactions, along with how they relate to the cardiovascular system and Kounis syndrome, which is hypersensitivity-associated. Considering all of the previously published information, we aim to discuss the clinical characteristics of specific crucial topics associated with Kounis syndrome, including immunoglobulin E and serum tryptase, common pathways of allergic and nonallergic cardiovascular events, current perspectives on Kounis syndrome, allergic angina, allergic myocardial infarction, and the impact of Coronavirus disease 2019 (COVID-19) and its vaccination on Kounis syndrome.

## 2. The Antecedents of Kounis Syndrome

**a. Foreign Proteins Inducing Acute Carditis**

One hundred years or so ago, experiments showed that the myocardium may alter in response to a foreign protein [3]. After receiving repeated intoxicating injections of foreign proteins, studies conducted on rabbits revealed large regions of focal degeneration of the heart muscle cells as well as the kidney and liver cells.

Sometimes a single injection of a foreign protein can cause interstitial myocarditis. Additionally, sensitized guinea pigs that received injections of foreign proteins developed lesions of the liver, kidney, spleen, and heart’s smaller arteries. These lesions were characterized by endothelium regeneration, media and intima edema, and occasionally by internal elastic lamina splitting. Around the smaller coronary arteries, additional experiments confirmed early findings by detecting perivascular granulomas, necrotic areas, cellular infiltration, and endothelial proliferation. Patients with pneumonia experienced severe serum reactions and myocarditis after receiving large amounts of serum. The necrotizing arteritis and periarterial inflammation revealed by necropsy showed a proliferation of histiocytes in the mural and valvular endocardium, the aortic and pulmonary artery intima, and the interstitial tissue of the myocardium, liver, kidneys, and adrenal glands. This was accompanied by focal edema of the surrounding tissue. The severe serum sickness was thought to be the cause of the pathologic findings in these patients, which resembled those caused by anaphylaxis in animals [2].

Horse serum administered intramuscularly and intraspinally to a young poliomyelitis patient resulted in a reaction that included lymphadenopathy, edema, and an erythematous rash. The results of the autopsy revealed localized infiltration between the muscle fibers and diffuse infiltration of histiocytes in the heart’s connective and subendocardial tissues. It is important to emphasize that histiocytes share common immunophenotypic and histological traits, as shown by immunostains. They have membrane receptors for opsonins, including complement fragment complement component 3 (C3b) and immunoglobulin G (IgG). They express cluster of differentiation antigens CD45, CD14, CD33, and CD4 (also expressed by T helper cells), which are leucocyte common antigens. They have varying numbers of lysosomes in their eosinophilic cytoplasm.

Therefore, histiocytes are components of the organism’s immune system, which includes the mononuclear phagocytic system. The histiocytes are either dendritic cells [10] or tissue macrophages [11]. When neutrophils have reached the end of their life, they play a part in eliminating them.

**b. Rheumatic carditis and Morphologic cardiac reactions**

Approximately one hundred years ago [12], it was proposed that the lesions of rheumatic fever might be caused by hypersensitivity reactions to bacterial products rather than by the direct action of a bacterial toxin of an invisible virus. Rheumatic fever can cause focal inflammatory involvement of the interstitial tissue in all three layers of the heart, a pathological condition known as pancarditis. Aschoff nodules or Aschoff bodies were the pathognomonic sign of pancarditis in rheumatic heart disease [13]. Research conducted at the time confirmed the idea that verrucose valvular vegetations, focal alterations in the collagen of the surrounding tissue, Ashoff body, diffuse and total inflammatory lesions, and focal alterations in the cardiac muscle were the hallmark cardiac lesions of rheumatic fever. The cardiac lesions associated with rheumatic fever were at least strongly suggested to be the consequence of anaphylactic hypersensitivity by these experimental findings and other factors [14]. Although more research was clearly needed to establish a solid understanding of the pathophysiology of human rheumatic fever, the authors, at that time, came to the conclusion that their findings might suggest worthwhile research directions. Their main goal was to highlight the fundamental similarities between cardiac lesions of known anaphylactic origin and those associated with carditis, using as many clear examples as possible [4].

## 3. Allergic Angina and Allergic Myocardial Infarction

Nearly 5 centuries had to pass until 1903 when in allergy experiments on dogs and rabbits, it was observed that allergy affects the heart both clinically and on electrocardiograms. In the year 1903, the Dutchman Einthoven built the first electrocardiograph [15], and after 21 years, he received the Nobel Prize in Physiology. What a coincidence! It is truly a mystery that allergy since then coincides and goes hand in hand with the discovery of the electrocardiograph.

The cardiovascular symptoms and signs had already been identified as being the main clinical manifestations associated with allergic or hypersensitivity and anaphylactic or anaphylactoid attacks. But it was not until 1950 that a 49-year-old man experienced urticaria and anteroseptal myocardial infarction following four days of treatment with 300,000 units of penicillin in oil; this condition was diagnosed as allergic myocardial infarction. Dicumarol, papaverine, morphine, and diphenhydramine hydrochloride were used to treat the patient, who was diagnosed with allergic myocardial infarction [16]. However, a new syndrome was first established in 1991 with the accidental co-occurrence of allergic reactions and chest pain, along with laboratory and clinical signs of classical angina pectoris brought on by inflammatory mediators released during the allergic insult. They called the condition allergic angina syndrome [17].

This clinical description led Constantinides to suggest in 1995 that common allergic reactions might encourage plaque disruption [18]. According to Brawnvald’s 1998 editorial, allergic reactions with mediators such as histamine or leukotrienes acting on coronary vascular smooth muscle can cause vasospastic angina [19].

These days, allergic angina and allergic myocardial infarction are coronary artery disorders that with other allergic vascular disorders can be brought on by a variety of constantly growing causes, as well as a wide range of mast cell-associated disorders that can affect patients of any age and cause multi-organ arterial system involvement along with a widening of clinical symptoms and signs.

## 4. Current Views on Kounis Syndrome

**Figure 1:**
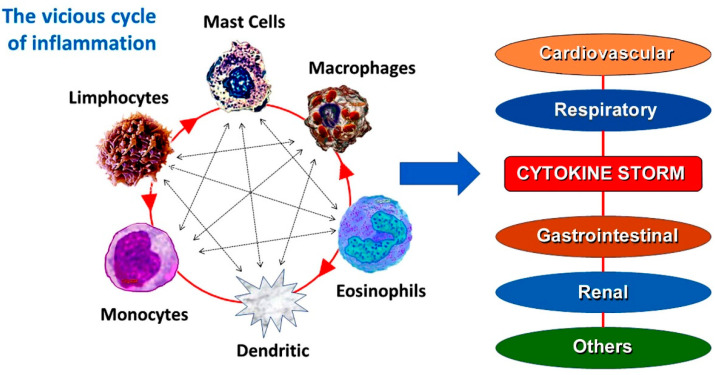
These inflammatory cells respond to various stimuli by activating one another like a ball of thread, creating a vicious cycle.

Today, Kounis syndrome is characterized as a distinct kind of acute vascular syndrome that affects not only the coronary arteries but also the cerebral [20,21,22,23], mesenteric [23,24], peripheral [25], and venous systems [26]. Moreover, Kounis syndrome is not a single-organ vascular disorder but a multisystem and multidisciplinary disease [27]. This syndrome is brought on by the inflammatory mediators that are released during an allergic insult by mast cell degranulation and other interacting cells, including T-lymphocytes, macrophages, eosinophils, and platelets [28] (Figure 1).

**Figure 2:**
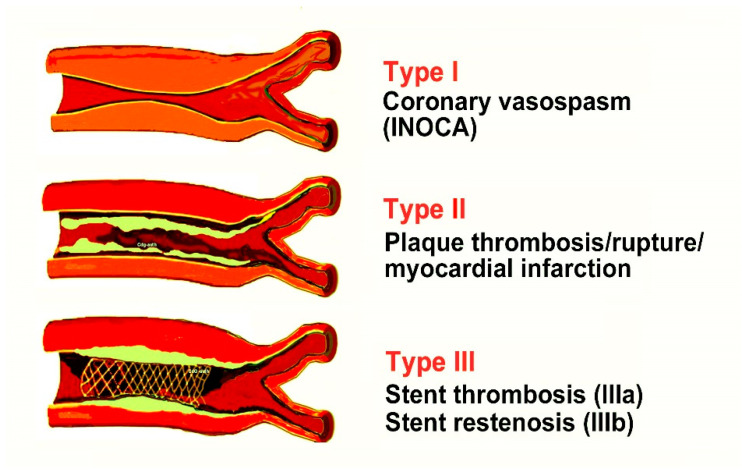
The types and subtypes of Kounis syndrome.

By inducing coronary spasm, atheromatous plaque erosion or rupture, and platelet activation in the Kounis syndrome cascade, chymase, tryptase, histamine, and arachidonic acid products can all contribute to the acute ischemic event. In patients who suffer an allergic, hypersensitive, anaphylactic, or anaphylactoid insult, its incidence varies between 1.1 and 3.4% [29]. Kounis syndrome was once thought to be a rare condition, but it appears to be underdiagnosed. Thus far, this syndrome has been classified into three types [30] (Figure 2).

Type I or MINOCA-type myocardial infarction with nonobstructive coronary arteries is caused by histamine, chymase, or arachidonic acid products (leukotrienes and platelet-activating factor) and affects 76.6% of patients with normal or nearly normal coronary arteries.

Type II is caused by the same factors as type I plus acute myocardial infarction with platelet activation and affects 22.3% of patients with quiescent preexisting coronary disease.

Type III is stent thrombosis (subtype IIIa) and/or stent restenosis (subtype IIIb), which affects 5.1% of patients, and is brought on by stent polymers, stent metals, eluted medications, dual antiplatelets, and environmental exposure.

## 5. Common Pathway Between Allergic and Not Allergic Vascular Events

Most cases of unstable angina and acute myocardial infarction are almost certainly caused by a combination of coronary artery spasm and atheromatous plaque erosion or rupture, which is followed by the formation of thrombus. Patients with acute coronary syndromes of nonallergic etiology have been found to have higher levels of the following mediators in their blood or urine, which are released during acute allergic episodes [31]. Individuals with nonallergic acute coronary syndromes have been found to have blood histamine concentrations that are more than twice as high as those of healthy individuals. The biogenic amine histamine is primarily present in certain histaminergic neurons and mast cells. Histamine uses four G-protein-coupled receptors, numbered one through four, to carry out its numerous and diverse effects [32]. In the acute stage of nonallergic myocardial infarction, arachidonic acid metabolites like thromboxane and leukotrienes have been discovered to be substantially more prevalent in the systemic arterial circulation compared to the circulation of normal controls [33]. Patients with nonallergic acute coronary syndromes have been shown to have higher levels of interleukin-6, which is produced from inflammatory coronary plaques and regions of myocardial necrosis [34,35]. Examples of nonallergic post-mortem acute myocardial infarction were found to share a common pathway between allergic and nonallergic coronary events. Researchers have found a significantly higher (200:1) degree of mast cell degranulation at plaque erosion or rupture sites compared to adjacent regions or even farther-flung segments. The question in this report was how, considering that mast cell maturation and mediator release can take days or weeks, such high concentrations of degranulated mast cells were found at the plaque erosion or rupture sites [36]. It seems likely that the mature mast cells would have been ready to degranulate and release their contents at the erosion or rupture sites before the acute coronary event. There are important therapeutic and clinical implications for preventing coronary plaques and the progression of unstable lesions by inhibiting mast cell degranulation and the ensuing acute myocardial infarction. Actually, monoclonal antibodies that reduce IgE receptors in the mast surface and drugs and substances that stabilize the mast cell membrane could be considered novel therapeutic strategies to prevent acute coronary and cerebrovascular disorders [37]. A common pathway between allergic and nonallergic coronary events was found in cases of nonallergic post-mortem acute myocardial infarction.

## 6. Myocardial Infarction, a Preventable Disease?

Kounis syndrome and nonallergic coronary events seem to be produced via a similar mechanism. Therefore, is Kounis syndrome a magnificent example of nature’s own experiment and a final trigger pathway linked to plaque rupture and coronary artery spasm? So far, the following actions could be taken to avoid myocardial infarction from other causes as well as Kounis syndrome [38].

Mast cell degranulations can be inhibited by a receptor on mast cells MRGPRX2. This receptor mediates IgE-independent degranulation and has been linked to a number of disorders mediated by mast cells [39].

Mast cell activation can be prevented by using transmembrane SCF embedded in lipid nanodiscs or proteoliposomes. This is because the toxicity linked to mast cell activation has limited the use of drugs that target SCF. These treatments can provide therapeutic and preventative benefits without producing adverse effects [40].

Sodium cromoglycate stabilizes the mast cell membrane, and dexamethasone has been used to lessen inflammation. Late thrombotic events have been avoided when these two agents are used together [41].

In order to treat IgE-mediated coronary syndromes, humanized monoclonal antibodies, like omalizumab against IgE antibodies, have already been effectively used to treat recurrent Kounis syndrome [37].

The identification of Kounis syndrome demonstrated that the same inflammatory mediators, which are released by the same inflammatory cells, are found in the bloodstream and during nonallergic coronary events.

## 7. IgEs and Kounis Syndrome

The complex interaction of genes, cytokines, and exposure to environmental antigens results in the production and release of IgE antibodies by B lymphocytes.

As part of a protein network involved in signaling response to antigens and allergens, IgE antibodies play a role in atopic diseases and systemic anaphylaxis [42]. Although IgEs only last a day in plasma, they can bind to receptors and stay attached to cells in tissues for weeks or months. Their biologic activity primarily depends on their ability to bind to particular fragment crystallizable region (FcR) receptors, primarily found on the surface of mast cells but also of basophils, eosinophils, monocytes, and epithelial and dendritic cells. These receptors are low-affinity fragment crystallizable epsilon region ΙΙ (FcεRII) and fragment crystallizable gamma region ΙΙ (FcgRII) and high-affinity fragment crystallizable epsilon region Ι (FcεRI) and fragment crystallizable gamma region Ι (FcgRI). The above receptors are upregulated and cell survival is increased when IgE binds to FcR [43]. IgEs bound to mast cells interact with corresponding antigens or allergens on the mast cell surface to cause mast cell degranulation and the release of a range of mediatory substances, both newly formed and preformed.

On the surface of mast cells or basophils, allergens cross-bridge their corresponding, receptor-bound immunoglobulin IgE antibodies to initiate allergic inflammation. When there are 2000 or more bridged IgE antibodies on the cell surface, out of a maximum of 500,000–1,000,000 IgE antibodies, these cells degranulate and release their mediators. A minimum of 1000 bridges are required to induce mast cell degranulation [44]. Independent of the presence of underlying atherosclerotic disease, mediator release can cause coronary artery spasm, which can present as myocardial infarction with nonobstructive coronary arteries (MINOCA) and Kounis type I syndrome [37]. The mediators may play a role in the atherosclerotic plaque’s critical progression, erosion, and rupture, causing thrombotic complications due to platelet activation and impairment of the fibrinolytic system, further encouraging the growth and development of arterial aneurysms [45]. In addition to upregulating IgE receptors, IgEs also promote mast cell survival, improve B cells’ ability to absorb allergens for antigen presentation, cause mast cells to express T-helper 2 cytokines, and amplify and prolong allergic reactions. Allergies, infections, and other immune disorders, such as hyper-IgE syndrome [46], a rare primary immunodeficiency disease marked by recurrent skin and pulmonary abscesses and extremely elevated IgE serum levels, can all result in elevated serum IgE. Furthermore, IgE levels may be elevated in both stable and unstable angina, acute myocardial infarction, and further correlate with the severity of acute myocardial infarction and plaque destabilization 1.8. Elevated IgE levels may also be a risk factor for increased cardiovascular mortality. It appears that elevated IgE levels occur prior to the coronary event and are not caused by an inflammatory response to tissue damage that occurs during the event. Indeed, according to a previous study [47], the levels of IgE and other immunoglobulin classes, including IgA and IgG, were noticeably higher prior to myocardial infarction in 270 initially healthy hyperlipidemic men than in matched healthy control subjects.

## 8. Lower IgEs Are Better for Kounis Syndrome

Patients with multi-vessel disease have substantially higher levels of total serum IgE than patients with single-vessel disease. They help distinguish the severity of coronary artery disease apart from conventional cardiovascular risk factors and are independent predictors of an elevated risk of multi-vessel disease. Serum IgE levels are elevated during acute coronary syndromes [42], according to a number of studies, which highlights the importance of monitoring these levels in patients who have experienced coronary events. As a result, the higher IgE levels seen in cardiovascular disease patients that occur prior to the coronary event [18] and their correlation with ischemic heart disease may be an early indicator of atherogenesis and its effects, which may help predict subsequent coronary events.

Humanized monoclonal anti-IgE antibodies attach to the constant region of IgE antibody molecules and stop IgE from attaching to low-affinity receptors, FcεRII, which are expressed on B cells, dendritic cells, and intestinal epithelial cells, and high-affinity receptors, FcεRI, which are expressed on the surface of mast cells and basophils. There is no chance of an allergic reaction right after the anti-IgE injection because anti-IgE cannot interact with IgE molecules once they are attached to IgE receptors. As a result, it cannot cross-link IgE and cause mast cell or basophil degranulation. The reduced expression of high-affinity IgE receptors FcεRI on mast cells and basophils, as well as a decrease in the release of histamine and other inflammatory mediators, is linked to the reduction in free IgE molecules brought on by anti-IgE therapy [48].

Omalizumab (anti-IgE) and mepolizumab (anti-interleukin-5 [IL5]), two important monoclonal antibodies, are being researched and used in relation to allergic coronary events. The humanized monoclonal anti-IgE antibody omalizumab, which is currently in use, bonds to the constant region (cε3) of the IgE molecule and stops free IgE from interacting with IgE receptors FcεRI and FcεRII, lowering the levels of circulating IgE. No matter how specific an allergen is, omalizumab will block the responses it causes because it does not attach to the variable allergen-specific region of the IgE molecule. By not binding to cell-bound IgE, omalizumab prevents the FcεRI cross-linking that may cause allergy, hypersensitivity, or anaphylaxis [49].

**Figure 3:**
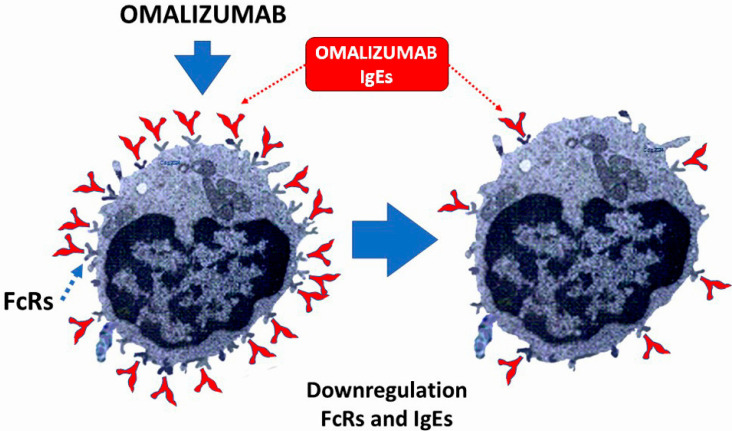
Reducing IgEs can help to avoid allergic myocardial infarction and allergic angina, and it also explains why all patients who have an allergic reaction do not develop Kounis syndrome.

Therefore, lowering IgEs can protect individuals who are at risk of developing allergic myocardial infarction and allergic angina. Additionally, the following appears to be the answer to the question of why all patients who experience an allergic reaction do not also experience Kounis syndrome: because their blood’s levels of IgEs are decreased (Figure 3).

## 9. Serum Tryptase, a Unique Mast Cell-Derived Cytokine

Tryptases chymases and cathepsins G are the major proteins stored and secreted by mast cells (Table 1). Tryptase is exclusively found in mast cells, though human basophils contain very small amounts of it (0.04 pg per basophil) [50]. Tryptases are expressed by tissue mast cells in healthy people, and they have been demonstrated to increase dramatically during anaphylaxis [51]. A higher baseline tryptase level may be a sign of mastocytosis, hereditary alpha-tryptasemia, and other nonallergic conditions. Higher baseline tryptase levels increase the risk of severe reactions in patients, particularly those with insect venom allergies, necessitating longer treatment. Serum acute tryptase and baseline tryptases must correlate for all acute systemic hypersensitivity reactions. The current diagnostic standard for verifying mast cell activation and anaphylaxis is measuring tryptase and detecting an increase from a basal serum tryptase level of more than 20% + 2 ng/mL (20 + 2 rule) when measured, within roughly 4 h of symptom onset [52]. A formula that has been validated in the perioperative setting, (2 + 1.2 × baseline tryptase level) [53], establishes the minimum acute elevation in the tryptase level required to demonstrate clinical significance [54]. Tryptase has a short half-life of roughly 90 min, just like other inflammatory mediators. The immunoreactive form of tryptase is stable, but it has a half-life of less than three minutes in physiological buffers. The aforementioned has led to the suggestion that the best time to obtain samples for tryptase determinations is 1–2 h after the precipitating event, but elevated levels of tryptase may be present in the circulation for several hours depending on the magnitude of the initial response [55].

Tryptase levels should only be measured one to two hours after the onset of symptoms in patients who have had myocardial infarction because they do not exhibit signs of an initial anaphylactic reaction with high tryptase release. Mast cells appear to undergo ultrastructural changes in their electron-dense granules, indicative of secretion in the second process of piecemeal degranulation, intragranular activation, or differential or selective release, but without overt degranulation. As a result, the amount of tryptase, if any, is insufficient to be detected by measuring the tryptase concentration in the systemic circulation [56].

Some patients with Kounis syndrome may have normal tryptase levels or undetected specific IgE, which could be better explained by the role played by non-IgE-dependent pathways that cause mast cell degranulation, such as MRGPRX2 on mast cells that can be activated by insect venoms and small-molecule antibiotics [57]. Thus, it is crucial that all patients with suspected Kounis syndrome have their mast cell tryptase levels measured. Asymptomatic controls (including those who are hereditary alpha tryptasemia (HαT): HαT-positive) typically have basal tryptase levels between 1 and 15 ng/mL. Overinterpretation, pointless referrals, and needless worry or anticipatory fear of illness in healthy people should all be avoided with this definition [58].

## 10. Kounis Syndrome, COVID-19, and Vaccines

**a. Actual Kounis syndrome and COVID-19**

Even though Kounis syndrome—an acute coronary syndrome brought on by inflammatory stimuli—is a relatively non-frequent clinical condition, careful research and clarification are necessary to determine any possible interactions between COVID-19 and Kounis syndrome. An increased risk may result from pre-existing cardiovascular conditions like valvular heart disease, heart failure, or coronary artery disease.

A history of atopic or allergic diseases, such as hay fever, eczema, or asthma, may increase the risk of hypersensitivity reactions, which could lead to Kounis syndrome. More severe COVID-19 clinical manifestations have been linked to comorbid conditions like diabetes mellitus, hypertension, and obesity. One important possible mechanism is the cytokine storm and abnormally high levels of inflammatory mediators, such as C-reactive protein, tumor necrosis factor-alpha, and IL-6, seen in COVID-19 patients [59].

Stent thrombosis, myocardial infarction, and vascular spasm are symptoms of Kounis syndrome. The activation of mast cells and interactions between inflammatory cells, such as T-lymphocytes and macrophages, are linked to it. It is also linked to allergic, hypersensitive, or anaphylactic insults [60,61,62].

These cells leave the bone marrow as mononuclear cell precursors and enter the bloodstream as mast cell precursors, releasing SCF through their surface KIT receptors, which are cytokine receptors [9]. Growth, survival, differentiation, proliferation, adhesion, and homing of mast cells all depend on SCF. Since IgE antibodies are unable to penetrate the blood–brain barrier, mast cells can adhere to any human tissue, including brain tissue that is immune to allergic reactions. Mast cells in these tissues take days or even weeks to differentiate and mature. Different phenotypes are produced by the maturation of mast cells in the coronary arteries as a result of local microenvironmental factors [63].

The following evidence suggests and validates the idea that Kounis syndrome and COVID-19 are related:

1. Patients with COVID-19 exhibit cytokine storm and abnormally high levels of inflammatory mediators, including IL-6, tumor necrosis factor-alpha (TNF-a), and C-reactive protein (CRP). Because these inflammatory mediators can cause coronary vasospasm, plaque destabilization, and thrombus formation, they have been linked to the pathophysiology of Kounis syndrome [64].

2. Cytokines produced by mast cells, which are the main pathophysiological causes of Kounis syndrome, can increase the blood–brain barriers’ permeability, which accounts for the SARS-CoV-2 “COVID-19 brain fog”. This can happen either directly by activating mast cells or indirectly by allowing cytokines to enter through a compromised blood–brain barrier [65].

3. COVID-19 has an impact on the peripheral and coronary arteries. It may result in vascular or endothelial damage, hypoxic injury, cytokine storm, plaque rupture and microthrombi, coronary spasm, and an elevated risk of stent thrombosis. This is brought on by the underlying hypercoagulable disease, which clinically resembles the three primary forms of Kounis syndrome: stent thrombosis, coronary spasm, and acute myocardial infarction [29].

4. Activation of the immune system by COVID-19 in asymptomatic patients may raise the chance of developing Kounis syndrome, an unstable condition with susceptible plaques prone to thrombosis, from asymptomatic, subclinical, or atherosclerotic disease [66].

**b. COVID-19 vaccines and Kounis syndrome**

There are relatively few patients who have developed Kounis syndrome following COVID-19 vaccination in comparison to the total number of vaccinated patients, and further case–control and direct experimental testing is still required to confirm a conclusive link between vaccination and Kounis syndrome [67]. Even so, vaccination is still a promising option for vulnerable groups given the symptoms of COVID-19, even though there is a chance of rare side effects like Kounis syndrome. However, the Vaccine Adverse Event Reporting System received reports of 175 cases of severe allergic reactions from the 1,893,360 people who received their first dose of the Pfizer-BioNTech COVID-19 vaccine. Twenty-one of these were categorized as anaphylaxis. Seven people who had previously experienced anaphylaxis and seventeen people with a documented history of allergies or allergic reactions were among those who suffered anaphylaxis [68].

According to autopsy data, approximately half of deaths are caused by cardiovascular events, and the majority of deaths happen the same day or the day after COVID-19 mRNA-lipid nanoparticle vaccines are administered. Conventional vaccines usually cause these side effects to be ascribed to allergic reactions, but COVID-19 mRNA-LNP vaccines have been linked to a death rate from cytokine storms [69].

Worldwide, all COVID-19 vaccines have the potential to cause allergic reactions, particularly in atopic individuals. Some of the excipients used in the COVID-19 vaccine have been suspected of being the culprits. Indeed, Pfizer-BioNTech mRNA COVID-19 vaccines contain the excipient polyethylene glycol, the viral vector Covishield vaccine which is made in India and resembles the AstraZeneca vaccine, contains aluminum hydroxide, polysorbate 80, and disodium edetate dihydrate (ethylenediaminetetraacetic acid). Trometamol, also referred to as tromethamine, and polyethylene glycol are ingredients in the Moderna vaccine. Tromethamine constitutes an excipient of gadolinium-based contrast agents. There is polysorbate 80 in the Johnson & Johnson vaccine. Disodium ethylenediaminetetraacetic acid dehydrate and polysorbate 80 are components of the Sputnik V vaccine. Sinovac (Coronavac), a Chinese-made product, contains sodium chloride, sodium dihydrogen phosphate monohydrate, and disodium hydrogen phosphate [70]. All of these excipients may cause sensitization in their users and are also present in creams, ointments, lotions, other cosmetics, dental materials, laxatives, and anticancer medications. Between 1 and 5.4% of people are thought to already be sensitized to cosmetics or their ingredients. Oncology drugs containing free polysorbate are already available [71]. Alkyl saccharides, which can decrease immunogenicity, enhance stability, inhibit oxidative damage issues, and possibly avert thrombotic and cardiovascular events, are promising substitutes for these excipients [72,73].

**Perspective**

Up until now, researchers have been able to prevent late thrombotic events by using sodium cromoglycate to stabilize the mast cell membrane and dexamethasone to reduce inflammation. Furthermore, humanized monoclonal antibodies, transmembrane stem cell factor (SCF) embedded in lipid nanodiscs or proteoliposomes, and mass-related G-protein-coupled receptor X2 (MRGPRX2) could be used to prevent Kounis syndrome and myocardial infarction from other causes. Therefore, is Kounis syndrome a magnificent example of nature’s own experiment and a final trigger pathway linked to plaque rupture and coronary artery spasm? It is unclear if this question will be addressed in upcoming clinical trials. If this is the case, atopic patients who are susceptible to food drug and environmental exposure-induced allergies as well as those who are likely to develop Kounis syndrome may benefit from the selective stabilization and protection of the mast cell surface membrane.

## Figures and Tables

**Table 1:** The activities of mast cell secretory granules released endopeptidases known as tryptases, chymases, and cathepsins.

Tryptase	Chymase	Cathepsin D
Activates the zymogen forms of metalloproteinases such as interstitial collagenase, gelatinase, and stromelysin and can promote plaque disruption or rupture	Converts angiotensin Ι to angiotensin II. Angiotensin II receptors are found in the medial muscle cells of human coronary arteries. Thus, angiotensin II generated by chymase could act synergistically with histamine and aggravate the local spasm of the infracted coronary artery. Chymase can also remove cholesterol from HDL	Angiotensin II-forming protease
Degrades the pericellular matrix components fibronectin and vitronectin and neuropeptides such as vasoactive intestinal peptide and calcitonin gene-related peptide	Activates matrix metalloproteinases 1, 2, 9 and plays a major role in the physiologic degradation of fibronectin and thrombin	Degrades both fibronectin and vascular endothelial cadherin, which are necessary for the adhesion of endothelial cells to their basement membrane and to each other
Tryptase can degrade high-density lipoprotein, (“good” cholesterol)		
Activates neighboring cells by cleaving and activating protease-activated receptor (PAR)-2 and thrombin receptors		

## Data Availability

No new data were created or analyzed in this study. Data sharing is not applicable to this article.

## Abbreviations

C3b	Complement component 3
COVID-19	Coronavirus disease 2019
CRP	C-reactive protein
FcR	Fragment crystallizable region
F FcεRI	Fragment crystallizable epsilon region RI
FcεRII	Fragment crystallizable epsilon region RII
FcgRI	Fragment crystallizable gamma region RΙ
FcgRII	Fragment crystallizable gamma region ΙI
IgE	Immunoglobulin E
Ι IL-6	Interleukin-6
IgE	Immunoglobulin G
SCF	Stem cell factor
TNFα	Tumor necrosis factor-alpha
